# Segmentation of Bone with Region Based Active Contour Model in PD Weighted MR Images of Shoulder

**DOI:** 10.1155/2015/754894

**Published:** 2015-05-07

**Authors:** Aysun Sezer, Hasan Basri Sezer, Songul Albayrak

**Affiliations:** ^1^Computer Engineering Department, Yildiz Technical University, 34220 Istanbul, Turkey; ^2^Orthopaedic and Traumatology Clinic, Sisli Hamidiye Etfal Training and Research Hospital, 34360 Istanbul, Turkey

## Abstract

Proton density (PD) weighted MR images present inhomogeneity problem, low signal to noise ratio (SNR) and cannot define bone borders clearly. Segmentation of PD weighted images is hampered with these properties of PD weighted images which even limit the visual inspection. The purpose of this study is to determine the effectiveness of segmentation of humeral head from axial PD MR images with active contour without edge (ACWE) model. We included 219 images from our original data set. We extended the use of speckle reducing anisotropic diffusion (SRAD) in PD MR images by estimation of standard deviation of noise (SDN) from ROI. To overcome the problem of initialization of the initial contour of these region based methods, the location of the initial contour was automatically determined with use of circular Hough transform. For comparison, signed pressure force (SPF), fuzzy C-means, and Gaussian mixture models were applied and segmentation results of all four methods were also compared with the manual segmentation results of an expert. Experimental results on our own database show promising results. This is the first study in the literature to segment normal and pathological humeral heads from PD weighted MR images.

## 1. Introduction

Shoulder instability constitutes an important mass of the shoulder surgery in orthopedics. Shoulder joint has a very wide range of motion and it is susceptible to instability because of less congruent bony relations. Shoulder instability is a condition in which relation between glenoid and humeral head is lost in a position or under physiological joint reaction forces. Instability may cause joint dysfunction and may result in dislocation of the joint which may cause further soft tissue and bone damage making disruption of anatomical structures worse. Instability of shoulder joint is a result of disruption in bony structures or soft tissues.

MRI is widely used to detect shoulder pathologies because it is able to demonstrate both bony and soft tissue pathologies which is essential to make accurate diagnosis. The humeral head being part of the bony structure of the shoulder joint plays an important role in shoulder instability.

Segmentation of bone has primary importance in order to define the anatomical borders and location of the lesions. Different imaging modalities are able to detect bony structures. Computerized Tomography (CT) and T1 weighted images were studied to be successful in bone segmentation in the literature. Pérez et al. used a hybrid, statistical, and geometrical model based on Chan-Vese to segment bone and soft tissues in T1 coronal shoulder MR images. They reported that their model provided correct classification of bone but poor classification of soft tissues [1]. Nguyen et al. studied sagittal T1 weighted shoulder images. They used an integrated region based and gradient based supervised method to segment humeral head. Their average success rate measured over entire database was 96.32% [2]. Chaoui et al. segmented bony structures by using a recognition based segmentation method from CT images with 96% success rate [3].

There is no single modality which can demonstrate bony edges and bone edema with high performance at the same time. CT and T1 weighted MR images are more successful in demonstrating bone edges. Transition between soft tissues and bone is sharper in CT and T1 weighted MRI. However these modalities cannot supply sufficient information about bone edema. Axial PD weighted MRI of shoulder is the mostly used sequence being able to present both bony and soft tissue components of instability synchronously. PD weighted MRI is also able to demonstrate bony edema which is a sign of the magnitude and location of bone trauma and a common source of pain. However there is a very smooth transition zone between bone and soft tissues, making segmentation of bone edges more difficult.

Another parameter that aggravates the segmentation process is noise. PD weighted MR images have poorer SNR than T1 weighted MR and CT images. Many researchers and numerous applications in medical images have been devoted to use nonlinear diffusion filters such as anisotropic diffusion (AD) and speckle reducing anisotropic diffusion (SRAD) to reduce noise while preserving image edges [4–6]. AD method has been applied to reduce noise in CT [7] MR [8] and ultrasound images with good results. AD method encourages diffusion in the homogeneous region while inhibiting diffusion at edges. However it is not directional and leaves some noise in the neighborhood of the edges.

SRAD method is directional that it inhibits smoothing in directions perpendicular to edges and encourages smoothing parallel to the edges not leaving noise in the vicinity of edges. SRAD was introduced as a useful technique to reduce speckle noise in ultrasound and satellite images successfully [4, 6]. Not only speckle noise but also other types of noise can be reduced by SRAD in case the standard deviation of noise (SDN) is estimated [9].

There is a wide variety of methods proposed to segment background and the object pixels among which the active contour model (ACM) was one of the most successful methods. The basic idea of the ACM is to evolve a curve under some constraints to extract the desired object based on energy minimizing method. Existing AC methods for image segmentation can be categorized into two classes: edge based models [10, 11] and region based models [12–15].

Edge based models utilize image gradient as an additional constraint to construct force to direct the motion of the contour. These models usually have edge based stopping constraint to intercept the contour evolution on the object boundaries [10]. High noise and weak or deficient borders of humeral head in PD images make edge based methods less suitable.

Region based active contour methods have many advantages over the edge based methods. First the region based methods do not depend on the image gradient and utilize image statistical information inside and outside the contour to control evolution. They can satisfactorily segment images with weak edges and without edges. Second, the segmentation result is less dependent on the location of the initial contour and they can effectively detect the exterior and interior boundaries simultaneously. Chan-Vese model is one of the most popular region based models which is based on a simplified Mumford-Shah segmentation techniques [12]. Chan-Vese model has been successfully applied to binary phase segmentation with assumption that each image region is statistically homogeneous. This assumption is a limitation of its application in inhomogeneous regions. In order to solve this limitation of Chan-Vese model, Vese and Chan [14] and Tsai et al. [15] proposed piecewise smooth (PS) models which can deal with part of the problem [12]. However, PS models have high computational costs. Moreover segmentation result is still dependent on the initial contour placement.

Li at al. proposed a local binary fitting (LBF) model to solve the problem caused by intensity inhomogeneity [16]. LBF model uses local statistical information, especially the local intensity mean, in a region based active contour model. However the intensity inhomogeneity problem cannot be solved with using particular uniform distribution because intensity nonuniformity in a desired object can show variations in different positions. Use of more than one kind of specific distribution is needed to describe the variation of intensity nonuniformity in each region. To solve this problem Ni et al. proposed control of evolution of contour by using the histogram of the intensity; however they experienced limitations in segmentation of natural images [17, 18]. Ge et al. defined a new regularization term with the anisotropic diffusion process based on the structure tensor and the duality theory. They computed the statistical information of magnitude of gradient to approximate the variation of the intensity by the anisotropic diffusion process [13]. Hybrid region based active contour (HRBAC) models were proposed recently to segment images with intensity inhomogeneity [19, 20]. HRBAC models, like SPF model [21] and geodesic intensity fitting model [22], combine merits of the traditional geodesic active contour (GAC) model, which is an edge based active contour model, and region based Chan-Vese model. Despite all of these models which were intended to solve the intensity inhomogeneity problem it is yet to be solved.

The common problem of all these methods is sensitivity to initialization. The problem of initial contour was studied by Wang et al. They proposed to combine the local and global intensity information [23]. The limitation of the study was the deviation of the real object boundary when the initial contour was close to the object boundaries. Liu and Peng proposed to use degraded Chan-Vese model, the segmentation result of which is taken as initial contour of proposed local region based Chan-Vese model. Their model can segment images with intensity inhomogeneity; although this method is computationally efficient, the success rates are still sensitive to the initial contour [24].

In this paper we studied humeral head (bone) segmentation in axial PD MRI of shoulder. In the first step we applied SRAD method and homomorphic filter. SRAD is very sensitive to the estimation of SDN and normally calculated in a homogenous area [4, 9]. We estimated this parameter from area of interest where there is more tissue intensity than background to reduce Rician noise. By this way we extended use of SRAD in PD MR images. Noise reduction with SRAD makes a good contribution to the correction of inhomogeneity because the image noise is a factor that affects quantity of inhomogeneity. But noise reduction with SRAD alone is not enough for satisfactory correction of inhomogeneity in the image. Thus we supported SRAD method with homomorphic filter. This filter separates low and high frequency components in an image and enhances changes in high frequency component and suppresses changes in low frequency component to obtain more homogenous images. Moreover, the homomorphic filter is in its essences a linear filter and is quintessentially used for noise reduction or signal feature extraction if the signal (PD MR image) is distorted by additive noise.

Region based active contour models have ability to segment very weak edges of humeral head but they are sensitive to the location of initial circle. In the second step this problem was handled with the automatic detection of the initial contour in each shoulder image. We used circular Hough transform to automatically detect the location of the humeral head from PD weighted MR images. Result parameters obtained by circular Hough transform were used to identify the ROI and the place of initial contour.

In the last step we applied ACWE method [12] to segment humeral head in the ROI. We compared results of ACWE with SPF and clustering methods of fuzzy C-means (FCM) and Gaussian mixture model (GMM). ACWE has a global segmentation property and provides to segment all objects in the ROI. SPF has a local property which provides to only extract a desired object by setting the initial contour surrounding or intersecting the humeral head boundaries [21]. All segmentation results were compared with the manual segmentation results of an orthopedic specialist.

Note that some part of results of the segmentation of normal humeral heads in this paper was reported in our recent conference paper [25]. This paper is organized as follows. In Section 2 description of materials and methods, steps of preprocessing, segmentation, and postprocessing methods are described. Experimental results and evaluation are given in Section 3, followed by some discussion in Section 4. This paper is summarized in Section 5.

## 2. Material and Methods

MRI is a standard of protocol in evaluation of the shoulder instability. We used 2D PD weighted MR images of shoulder which can successfully demonstrate the pathologies that cause shoulder instability. MRI has the capacity to present 3D images; however pathological changes in shoulder instability may be hidden in 3D images. 3D MRI has no advantage over 2D MRI in demonstrating the whole pathology.

The humeral head is the part of the bony structure of the shoulder joint. The pathologies of humeral head in shoulder instability can be categorized as bone edema and disruption of bone borders (e.g., Hill-Sachs lesion). In this study we segmented normal humeral heads, humeral heads with bone edema, and Hill-Sachs lesion from 2D axial PD MR images.

**Figure 1:**
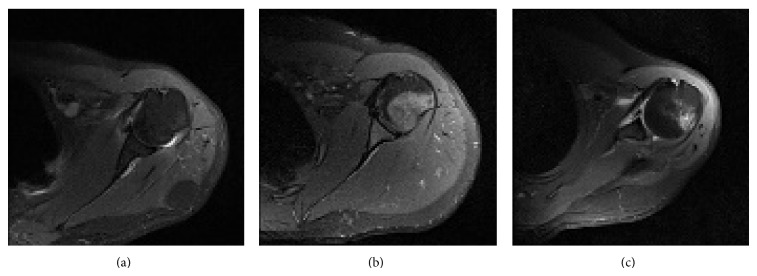
PD weighted axial MR images of left shoulder with (a) normal humeral head, (b) edematous humeral head, and (c) humeral head with Hill-Sachs lesion.

A normal humeral head image of left shoulder is shown in Figure 1(a). Humeral head with edema is demonstrated in Figure 1(b). The amount and the location of bone edema change according to patient. Hill-Sachs lesion occurs when the humeral head has a compression fracture where the distribution of intensity values increases and shape of humeral head changes (Figure 1(c)).

**Figure 2:**
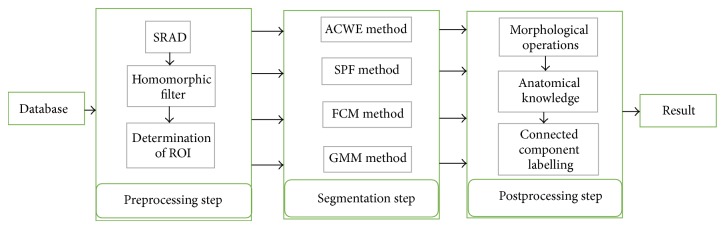
The flow chart of the segmentation steps by ACWE and the comparison methods of SPF, FCM, and GMM.

We included shoulder MR images of 219 patients who have been admitted to Sisli Hamidiye Etfal Training and Research Hospital. We used 1.5 Tesla PD weighted MR images. The size of the DICOM image was 256 × 256 pixels. The slice thickness was 4 millimeters. We grouped patients according to appearance of humeral head as normal, edematous, and Hill-Sachs deformity. The numbers of the patients were 81 in normal group, 100 in edematous group, and 38 in Hill-Sachs group. Segmentation of humeral head from PD weighted MR images was not studied in literature and there was no available database. The original data set was collected by authors. Segmentation process in this study may be categorized into three steps as preprocessing, segmentation, and postprocessing (Figure 2).

### 2.1. Steps of Image Preprocessing

#### 2.1.1. Noise Reduction Using Speckle Reducing Anisotropic Diffusion (SRAD)

MR images can be categorized into two classes: high resolution with high SNR and high resolution with low SNR. High resolution with high SNR is a consequence of increased acquisition time of MRI which is not feasible due to the patient comfort. Noise reduction in PD weighted MR images has an important effect on segmentation success as much as correction of intensity inhomogeneity. Owing to the fact that the distribution of the noise in PD images is not Gaussian, noise problem cannot be solved sufficiently by traditional noise reduction methods such as Gaussian filter and median filter [8].

**Figure 3:**
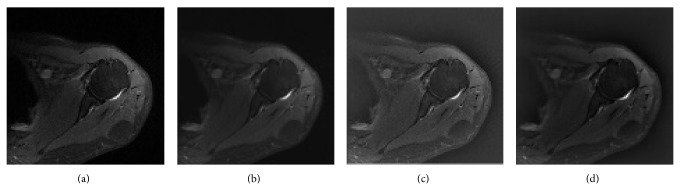
(a) Orginal PD weighted MRI, (b) image obtained after SRAD method, (c) result of the homomorphic filter, and (d) result obtained after using SRAD method and homomorphic filter.

PD weighted MR images have a very smooth transition zone between soft tissues and bone (Figure 3(a)). There is need of a method which decreases noise without deteriorating the important anatomical and pathological details of humeral head. AD method was proved to produce successful results in medical imaging area; however AD method leaves some remaining noise in the vicinity of edges after filtering [4, 7, 8]. We used SRAD which is a directional method that overcomes this problem by inhibiting smoothing parallel to edge and enhancing smoothing in perpendicular direction (Figure 3(b)).

SRAD method demonstrates good speckle noise suppression in ultrasound, satellite images, and MRI. The weak side of SRAD method is its sensitivity to the SDN. Through the accurate estimation of SDN SRAD method can be applied for all kinds of noise not only for speckle noise [9]. SRAD method is a combination of AD and speckle reducing Lee filter.

The partial differential equation (PDE) of AD is given as follows in continuous domain, where *I*_0_ is the initial image, *c*( ) is the diffusion coefficient, ∇ is gradient operator, and div is divergence operator:

(1) ∂I∂tdiv⁡c∇I∇I,

(2) It=0=I0.

We used SRAD to decrease noise in the PD weighted MR images. Given an intensity image *I*_0_(*x*, *y*) having finite power and no zero values over the image support domain *Ω*, the output image *I*(*x*, *y*; *t*) is evolved according to the following format of the PDE of SRAD [4], where *I*(*x*, *y*; *t*) is the intensity image estimated at position *x*, *y* at the diffusion time *t*; ∂*Ω* denotes the border of *Ω*; $\vec{n}$ is the outher normal to the ∂*Ω*:

(3) ∂Ix,y;t∂tdiv⁡cq∇Ix,y;t,

(4) Ix,y;0=I0x,y,∂Ix,y;t∂n→∂Ω=0.

SRAD uses the format of the PDE of AD. Selection of diffusion coefficient is the main difference between AD and SRAD methods ((1) and (3)):

(5) cq=11+q2x,y;t−q02t/q02t1+q02t.

SRAD inherits instantaneous coefficient of variation (ICOV) and speckle scale function prototype of Lee filter. ICOV serves as the edge detector. ICOV is a function of the image normalized gradient magnitude ∇*I*/*I* and normalized Laplacian ∇^2^ *I*/*I* defined in relation with the adaptive coefficient of the Lee filter [4] expressed as follows:

(6) $$\begin{array}{c} q\left(\;x,y;t\;\right)=\sqrt{\frac{\left(1/2\right){\left(\left|\nabla I\right|/I\right)}^{2}-\left(1/16\right){\left(\nabla^{2}I/I\right)}^{2}}{\left[1+\left(1/4\right){\left(\nabla^{2}I/I\right)}^{2}\right]}}. \end{array}$$

Speckle scale function *q*_0_(*t*) controls amount of smoothing applied to the image by SRAD and computed from a homogeneous region of fully developed speckle by

(7) $$\begin{array}{c} q_{0}\left(\;t\;\right)=\frac{\sqrt{\mathrm{var}\left[z\left(t\right)\right]}}{\overset{-}{z\left(t\right)}}. \end{array}$$

But we have calculated speckle scale function on the foreground where the humeral head is dominant than other tissues. This way, we assure that the background has no effect over the estimation of Rician noise present in MRI. The var[*z*(*t*)] and $\overset{-}{z(t)}$ are intensity variance and mean over humeral head region at time *t*.

#### 2.1.2. Homomorphic Filter

Despite progress in the scanner technology, MR images still have imperfections like low SNR, intensity inhomogeneity due to the bias field, and other artifacts. Intensity inhomogeneity adversely affects visual inspection and segmentation. Numerous methods have been introduced to eliminate intensity inhomogeneity in MR images [26]. Homomorphic filtering assumes that intensity inhomogeneity is a low frequency artifact that can be separated from high frequency signal by low pass filtering. Pérez et al. used homomorphic filter in the image preprocessing step to segment bone and soft tissues on T1 weighted MR images with good results [1].

According to homomorphic filtering model, each pixel value *f*(*x*, *y*) can be expressed as the product of a low frequency illumination component *i*(*x*, *y*) and a high frequency reflectance component *r*(*x*, *y*) as follows:

(8) $$\begin{array}{c} f\left(\;x,y\;\right)=i\left(\;x,y\;\right)r\left(\;x,y\;\right). \end{array}$$

The homomorphic system for image enhancement is based on the separation of independent low and high frequency components. To facilitate their separate processing by taking logarithmic transform of (8), thus

(9) gx,yln⁡⁡fx,y=ln⁡⁡ix,yrx,y=ln⁡⁡ix,y+ln⁡⁡rx,y.

Fourier transform is applied to (9), where *G*(*u*, *v*), *I*(*u*, *v*), and *R*(*u*, *v*) represent the Fourier transform of *g*(*x*, *y*), ln⁡(*i*(*x*, *y*)), and ln⁡(*r*(*x*, *y*)), respectively. Consider

(10) $$\begin{array}{c} G\left(\;u,v\;\right)=I\left(\;u,v\;\right)+R\left(\;u,v\;\right). \end{array}$$

Applying a high pass filter *H*(*u*, *v*) in the frequency domain to enhance changes in reflectance and suppressing changes in illumination are as follows:

(11) $$\begin{array}{c} \tilde{G}\left(\;u,v\;\right)=I\left(\;u,v\;\right)H\left(\;u,v\;\right)+R\left(\;u,v\;\right)H\left(\;u,v\;\right). \end{array}$$

We used a Gaussian high pass filter instead of Butterworth high pass filter normally used in homomorphic filtering to remove the illumination component. Inverse Fourier transform is applied to (11) to obtain $\tilde{g}(u,v)\;$ which represent the inverse Fourier transform of $\tilde{G}(u,v)$ :

(12) $$\begin{array}{c} \tilde{g}\left(\;u,v\;\right)=\tilde{i}\left(\;u,v\;\right)+\tilde{r}\left(\;u,v\;\right). \end{array}$$

Exponential form $\exp(\tilde{g}(u,v))$ is used to reverse logarithmic effect to obtain the image after enhancement.

Our strategy for correction of inhomogeneity is to use the homomorphic filter to eliminate intensity inhomogeneity in the preprocessing step just after noise reduction with SRAD method (Figures 3(c) and 3(d)). We combined advantages of SRAD and homomorphic filter before application of region based active contour models.

#### 2.1.3. Determination of Region of Interest (ROI)

**Figure 4:**
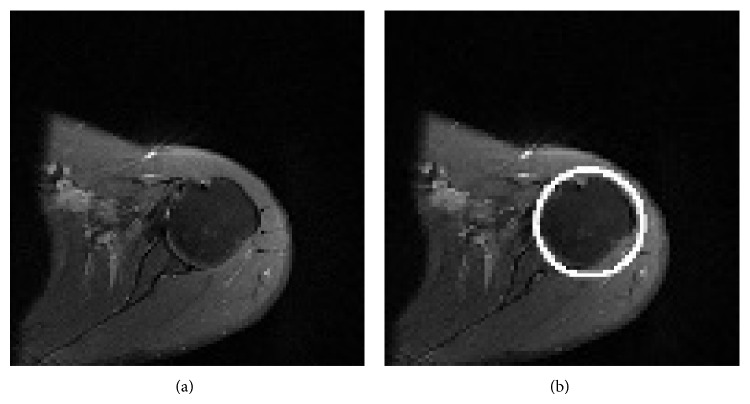
(a) Orginal MR image of left shoulder and (b) detection of humeral head by using the circular Hough transform.

The Hough transform is widely used in pattern recognition and computer vision for the detection of regular curves. Humeral head has a round cross-sectional shape in axial PD MR images. Circular Hough transform has an ability to detect circular shapes based on parametric equation of circle [27]. This method provides detection of humeral head for each PD weighted MR image without affecting image noise (Figure 4). This method is also very tolerant to gaps in feature boundary descriptions; thus it can also find defective shape of humeral head resulting from the edema and deformity.

Figure 4(b) shows detection of humeral head from a PD weighted left shoulder MR image. The shape of the humeral head is not exactly a circle; thus some parts of the area of interest may remain out of the circle defined by circular Hough transform. To involve whole humeral head in ROI we enlarged radius of circle found by circular Hough transform.

Common problem of region based active contour models is dependence of segmentation success on the initial contour placement [23, 24]. We achieved this problem by automatically determining location of initial contour in the ROI.

### 2.2. Segmentation Methods

#### 2.2.1. Active Contours without Edges (ACWE) Model

ACWE, known also as Chan-Vese model, is a region based active contour model and has successful applications in many papers and fields [1, 7, 12]. ACWE model utilizes statistical information inside and outside the contour instead of image gradient.

An evolving curve *C* = ∂*ω*, with *ω* ⊂ *Ω*  an open subset, inside (*C*) denotes the region *ω* which represents the foreground pixels and outside (*C*) denotes the region $\Omega \setminus \overset{-}{\omega }$ which represents the background pixels. Chan-Vese model is formulated by minimizing the following energy function with the level set formulation [12], (14):

(13) Fc1,c2,C=λ1∫Ωu0x,y−c12Hϕx,ydx dy+λ2∫Ωu0x,y−c221−Hϕx,ydx dy.

With the level set method we assume

(14) C∂ω=x,y∈Ω:ϕx,y=0,ωx,y∈Ω:ϕx,y>0,Ω∖ω−x,y∈Ω:ϕx,y<0.

The image *u*_0_ is assumed to be composed of two regions of constant intensities: *c*_1_ (the average intensities inside the contour) and *c*_2_ (the average intensities outside the contour). The constants *c*_1_ and *c*_2_ can be found by minimizing (13):

(15) c1ϕ∫Ωu0Hϕx,ydx dy∫ΩHϕx,ydx dy,

(16) c2ϕ=∫Ωu01−Hϕx,ydx dy∫Ω1−Hϕx,ydx dy.

Using the Heaviside function *H*(*ϕ*) and the Dirac function *δ*(*ϕ*) defined by Chan and Vese, the energy function can be expressed as follows:

(17) ∂φ∂t=δϕ·μ∇∇ϕ∇ϕ−v−λ11−c12+λ21−c22,

where *v* ≥ 0, *λ*_1_ ≥ 0, *λ*_2_ ≥ 0, and *μ* ≥ 0 are fixed parameters; *v* parameter is used to increase propagation speed; *λ*_1_ and *λ*_2_ parameters control the force inside and outside the contour; *μ* manipulates the smoothness of zero level set [12]. If *μ* value is large it detects only large objects while when *μ* value is small, small sized objects can also be detected.

ACWE model can satisfactorily segment a desired object having weak and deficient edges. This property of ACWE model matches up with our needs because PD images have a very poor transition between soft tissues and bones and some part of the border of bone is destroyed especially in images which has bone edema and cortical deformity. On the other hand PD images have a low SNR than other MR modalities which is also suitable for ACWE model which has ability to segment noisy images.

The weak points of this model are the assumption of each image region as homogeneous and sensitivity to initialization. Some related methods were proposed to solve intensity inhomogeneity problem [13, 16]. Some of them deal with part of the problem and to some extent they could perform segmentation of inhomogeneous objects. Our data set was composed of humeral heads presenting a wide morphological variance due to traumatic changes in borders and edema in the bone. Therefore the variance of the location and magnitude of bone and edema would render previously proposed methods less efficient in the humeral head segmentation from PD weighted MR images. Instead of correction of inhomogeneity during application of region based models we applied segmentation methods after intensity inhomogeneity was decreased with preprocessing. Besides, these methods cannot provide a reasonable solution to the initialization of the initial contour [24].

**Figure 5:**
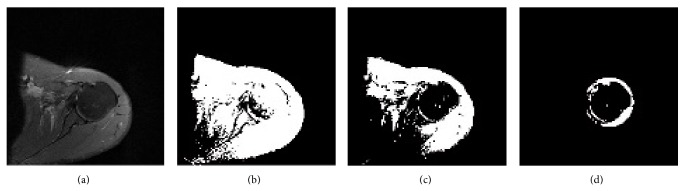
(a) Orginal PD weighted MR image of shoulder, (b) binary segmentation result of ACWE method without preprocessing operation, (c) segmentation result with ACWE after application of the SRAD and homomorphic filter, and (d) segmentation result of ACWE in ROI with the same preprocessing steps in (c).

When ACWE method was applied to the original PD weighted axial MR images segmentation of humeral head was notably unsatisfactory as shown in Figure 5(b). SRAD method was applied to decrease noise and homomorphic filter was used to obtain more homogeneous images. In the next step initial contour was automatically initialized in the humeral head. A better segmentation result was reached as shown in Figure 5(c). The humeral head was segmented from determined ROI with the same preprocessing step. As shown in Figure 5(d), segmentation success of humeral head was increased. Figure 5(d) also demonstrates some white pixels in the humeral head representing the edematous area of the humeral head.

#### 2.2.2. Signed Pressure Force (SPF) Model

SPF model is a combination of GAC [11] and region based Chan-Vese model [12] and possesses a local segmentation property. SPF model is able to segment selectively the desired object by setting initial contour intersecting or surrounding the desired boundaries. This model has advantages over GAC and Chan-Vese model. GAC model utilizes image gradient to construct an edge stopping function whereas SPF model uses statistical information to intercept contour evolution on the object boundaries. Zhang et al. stated that SPF method was capable of handling objects with weak or damaged boundaries. Furthermore they suggested that SPF model was able to segment objects with inhomogeneous interior intensity compared to Chan-Vese model [21]. In our data set some of the humeral heads had edema inside and weak or damaged surrounding edges. We applied SPF model to our data set and compared advantages of global segmentation property of ACWE method with local segmentation property of SPF.

SPF function was defined as follows:

(18) $$\begin{array}{c} \text{spf}\left(\;I\left(\;x\;\right)\;\right)=\frac{I\left(x\right)-\left(c_{1}+c_{2}\right)/2}{\max\left(\left|I\left(x\right)-\left(c_{1}+c_{2}\right)/2\right|\right)}, \end{array}$$

where *c*_1_ and *c*_2_ are defined in (15) and (16), respectively. The level set formulation of SPF model is as follows:

(19) ∂φ∂t=spfIx·∇ϕ∇ϕ+α∇ϕ+∇spfIx·∇ϕ,

where *α* is the balloon force term to shrink and expand the contour.

#### 2.2.3. Gaussian Mixture Model (GMM)

**Figure 6:**
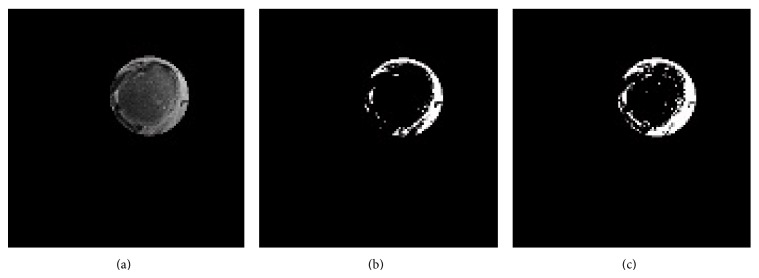
(a) Demonstration of orginal MR images of shoulder in determined ROI by circular Hough transform. (b) Segmentation result by using FCM method. (c) Segmenation result of GMM.

GMM is widely applied for segmentation in many fields. We used GMM clustering model to segment humeral head and the parameters of the model were estimated by using expectation maximization (EM) algorithm [28]. We applied this model in ROI defined by circular Hough transform (Figure 6(a)).

Let us observe image in a vector *xj*, *j* = 1,2,…, *n* and *i* ∈ {1,2,…, *k*}, and *k* is the number of regions. The mixture of Gaussian distribution is assumed as in the following form:

(20) $$\begin{array}{c} f\left(\;x\;\right)=\overset{k}{\underset{{i=1}^{\prime }}{\sum }}p_{i}N\left(\;x\;|\;\mu_{i},\sigma_{i}^{2}\;\right) \end{array}$$

*p*_*i*_ > 0 are weights such that ∑_*i*=1_^*k*^ *p*_*i*_ = 1:

(21) $$\begin{array}{c} N\left(\;\mu_{i},\sigma_{i}^{2}\;\right)=\frac{1}{\sigma \sqrt{2\pi }}\exp\left(\;\frac{{-\left(x-\mu_{i}\right)}^{2}}{2\sigma_{i}^{2}}\;\right). \end{array}$$

*Steps of EM-MAP Algorithm*. (1) Any classification method could be used to initialize parameter of *θ* ^(0)^. In our case we used *K*-means clustering method to define initial parameter of means (*μ*_*k*_^(0)^), variances *σ*_*k*_^(0)^, and weights *p*_*k*_^(0)^:

(22) $$\begin{array}{c} \theta^{\left(0\right)}=\left(\;p_{1}^{\left(0\right)},\ldots ,p_{k}^{\left(0\right)},\mu_{1}^{\left(0\right)},\ldots ,\mu_{k}^{\left(0\right)},\sigma_{1}^{\left(0\right)},\ldots ,\sigma_{k}^{\left(0\right)}\;\right). \end{array}$$

(2) E-step: *p*_*i*_^(*r*)^ is the discrete prior probability in stage *r* and *p*_*ij*_^(*r* + 1)^ is the discrete posterior probability in the next stage which is calculated by the Bayes rule as follows:

(23) $$\begin{array}{c} p_{ij}^{\left(r+1\right)}=p^{\left(r+1\right)}\left(\;i\;|\;x_{j}\;\right)=\frac{p_{i}^{\left(r\right)}N\left(x_{j}\;|\;\mu_{i}^{\left(r\right)},\sigma_{i}^{2\left(r\right)}\right)}{f\left(x_{j}\right)}. \end{array}$$

(3) M-step:

(24) p^ir+11n∑j=1npijr,μ^ir+1=∑j=1npijr+1xjnp^ir+1,σ^i2r+1=∑j=1npijr+1xj−μ^ir+12np^ir+1.

(4) E-step and M-step iterated until convergence to the arbitrary error:

(25) $$\begin{array}{c} \underset{i}{\sum }e_{i}^{2}<\varepsilon . \end{array}$$

GMM provides to segment normal humeral head images with two labels (Figure 6(c)).

#### 2.2.4. Fuzzy C-Means (FCM) Method

FCM method provides to assign pixels to each cluster based on minimizing the sum of distances from each pixel to every cluster centroid weighted by its corresponding membership. The objective cost function was minimized by assigning high membership values to pixels whose intensities are close to the centroid of its particular class and low membership values are assigned when the pixels are far from the centroid. Let *X* = (*x*_1_, *x*_2_,…, *x*_*N*_) represent an image with *N* pixels to be partitioned into *c* cluster [29]. The cost function is defined as follows:

(26) $$\begin{array}{c} J=\overset{N}{\underset{j=1}{\sum }}\;\overset{c}{\underset{i=1}{\sum }}u_{ij}^{m}{\left\|\;x_{j}-v_{i}\;\right\|}^{2}, \end{array}$$

where *u*_*ij*_ defines the membership of pixel *x*_*j*_ in the *i*th cluster and *v*_*i*_ is the cluster center. Fuzzy coefficient represented by *m* and *m* = 2 is used in this study. The membership function is updated iteratively by

(27) $$\begin{array}{c} u_{ij}=\frac{1}{\sum_{k=1}^{c}{\left(\left\|x_{j}-v_{i}\right\|/\left\|x_{j}-v_{k}\right\|\right)}^{2/\left(m-1\right)}}. \end{array}$$

Each cluster center is updated as follows:

(28) $$\begin{array}{c} v_{i}=\frac{\sum_{j=1}^{N}u_{ij}^{m}x_{j}}{\sum_{j=1}^{N}u_{ij}^{m}}. \end{array}$$

FCM model provides to segment normal humeral head images with two labels (Figure 6(b)).

### 2.3. Postprocessing

**Figure 7:**
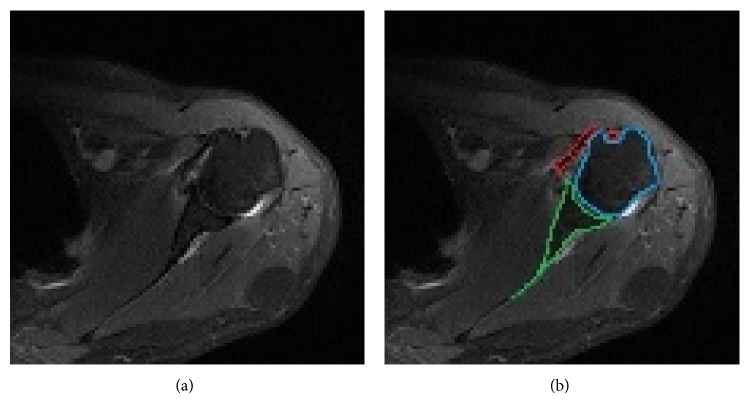
(a) Axial PD weighted MRI of shoulder; (b) biceps and subscapularis tendons are demonstrated in red area. The boundary of scapula and humeral head is colored in green and blue, respectively.

**Figure 8:**
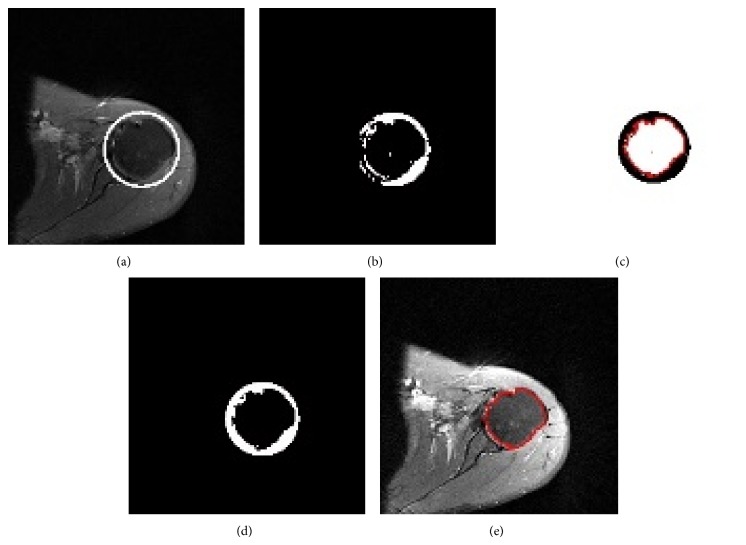
Steps of postprocessing. (a) ROI as a result of circular Hough transform. (b) The segmentation result in the ROI area. (c) The result of specific morphological operation and connected component labelling. (d) The filling of white pixels resulting from bone edema. (e) Segmentation result.

The segmented object after use of ACWE or SPF contains soft tissue components especially tendons which have a similar intensity value with humeral head (Figures 7(a) and 7(b)). After enlargement of the circle found with the circular Hough transform the scapular edge was also labeled as humeral head (Figures 8(a) and 8(b)). Scapula and some parts of tendons are located at the borders of the ROI circle. To eliminate scapula and tendon regions we applied specific morphological operations and connected component labeling method in the light of anatomical localization of tendons.

We determined the side of the humeral head by using histogram of an image. The scapula is located at the same direction with the shoulder side and in the periphery of the circle. According to the left and right shoulder images we may define the location of scapula area by using radius and center of circle which was obtained from circular Hough transform. In the scapula area we have done some erosion operations with circular structuring element. Connected component labeling method was used to detect connected regions in the complement of the binary image. Next the boundary of the humerus was labeled in red color (Figures 8(c), 8(d), and 8(e)). In the last step the white pixels resulting from the bone edema were filled to get the final result.

## 3. Experimental Results and Evaluation

Experiments were carried out with MATLAB 7.11 in Windows XP platform on a 2.5 GHz Intel (R) core (TM) personal computer with 4 GB of RAM. The manual segmentation tool was prepared in MATLAB environment and performed by using a mouse. To evaluate the success of segmentation we compared the segmentation results with the manually segmented areas determined by the orthopedic specialist. Similarity of segmented images is compared by Sorensen-Dice metric to find the success rates.

**Figure 9:**
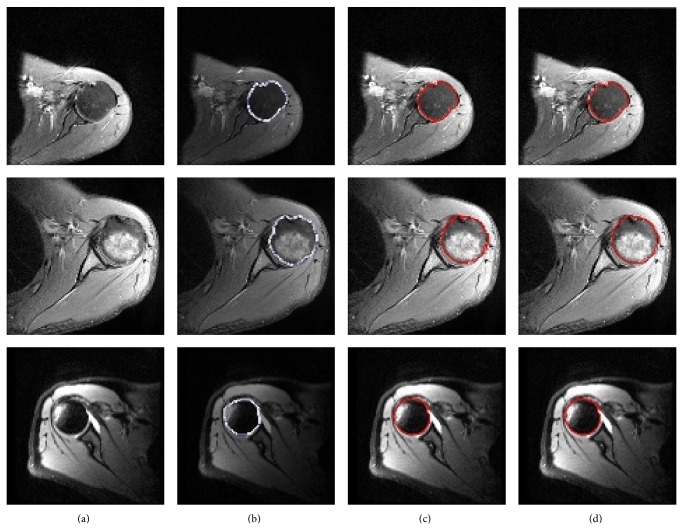
Segmentation results of normal, edematous humeral heads and humeral head with Hill-Sachs deformity were demonstrated in first, second, and third rows, respectively. Column (a) original images, (b) manual segmentation results by an expert, (c) segmentation results of ACWE method, and (d) segmentation results of SPF method.

We segmented normal and edematous humeral heads and humeral heads with Hill-Sachs deformity with ACWE and SPF methods (Figures 9(a), 9(b), 9(c), and 9(d)). The parameters used for ACWE are *μ* = 0.2, *λ*_1_ = 0.7, *λ*_2_ = 1, *v* = 1, and *ε* = 1.5. *λ*_1_ and *λ*_2_ parameters affect the uniformity and the resulting force inside and outside the contour, respectively. In our study we set *λ*_1_ < *λ*_2_ because we had uniform backgrounds and foreground containing varying grayscale objects. In order to detect smaller sized objects we assigned a small value to *μ* parameter. The parameters used for SPF method are *p* = 1, *σ* = 2, *K* = 5, and *ε* = 1.5.

We operated ACWE and SPF methods on determined ROI in 81 normal axial PD MR images. The average success rates of the ACWE and SPF before postprocessing according to Sorensen-Dice metric were 90.79% and 88.42%, respectively. After postprocessing operation segmentation results were increased to 92.17% in ACWE and 90.30% in SPF method as shown in Table 1.

We applied ACWE and SPF methods also in 100 edematous humeral heads and 38 humeral heads having Hill-Sachs deformity. The success rate of ACWE and SPF were 88.40–86.53% and 87.18–83.25% in edematous and defective humeral heads, respectively. Success rates of both of the methods increased approximately by 1-2% in edematous and defective humeral heads after postprocessing operation as shown in Table 1.

FCM and GMM were applied to whole data set; however they provided unsatisfactory results even with normal images (Table 2). As predicted, unsuccessful results were also obtained in edematous and defective humeral heads. FCM and GMM are vulnerable to pathologic changes in the humeral head like edema and Hill-Sachs deformity because of the changes in intensity of bone and blurred borders and deficient edges in these conditions.

## 4. Discussion

The ideal MRI study to evaluate shoulder instability is 2D axial PD slices in clinical studies. PD weighted images are gratifying in demonstration of bony edema that may help clinicians in the explanation of shoulder pain or the amount and place of trauma.

Segmentation of the humeral head from axial PD weighted shoulder MRI slices is complicated by four main reasons. The first and global reason is the innate feature PD weighted MRI which has a low SNR. The other important limitation is the soft transition between bony humeral head and surrounding soft tissues. The third difficulty results from the additional trauma to the humeral head that may cause bone edema in the ROI in variable amount and distribution. Changing amount and distribution of edema aggravates intensity inhomogeneity problem. The fourth issue that may hassle bony segmentation is the change of the shape of the humeral head after dislocation (Hill-Sachs lesion). These difficulties may also have adverse effects on visual inspection.

We recognized that without preprocessing step ACWE and SPF were unsuccessful in the entire PD MR images. In the image processing the image noise is a factor that affects inhomogeneity quantity. SRAD method is preferable because it decreases noise while protecting weak edges of humeral head. Application of SRAD to the Rician noise in a PD weighted MR image was carried out by estimating SDN from foreground. The use of homomorphic filter after SRAD further decreases inhomogeneity. Even though the decrease in the intensity inhomogeneity and the reduction of noise cannot be detected easily with pure inspection (Figures 3(b) and 3(c)), there is a remarkable and visually inspectable progress in the results of segmentation of preprocessed images when compared to the unprocessed images as shown in Figures 5(b) and 5(c). After noise reduction and decreased inhomogeneity in PD images ACWE and SPF would be estimated to work on PD weighted images.

The problem of the initialization of the initial contour is another issue to be solved to reach the desired success rates. ACWE and SPF can be operated with high success rates for segmentation of round cross-sectional objects with predetermination of the location of the initial contour automatically with circular Hough transform.

The most important obstacle affecting the success of the segmentation was the soft tissues (biceps and subscapularis tendons) and the remaining of scapula in the neighborhood of humeral head. It is very hard to eliminate the tendon tissues from the humeral head because of the similarity of their intensities in PD weighted images. In the postprocessing step we eliminated surrounding tissues with a specific morphological operation and using the knowledge of the anatomical structures.

In case of edema the intensity of the bone area increases and the borders of bone area blur. Deficient edges have additive effect on hampering the segmentation process. The overall results of segmentation in edematous and defective heads were lower than normal group by 1.97%–3.27% for ACWE method and by 0.98%–5.61% for SPF method, respectively. Moreover edema of the humeral head was labeled in white causing white pixels in the segmented humeral head resembling oversegmentation. Even in the humeral heads selected as visually normal there may be some minor edema which is hard to detect by visual inspection causing small areas of white pixels (Figure 5(d)).

Although in some cases like in Figures 9(c) and 9(d) there is not a visually inspectable difference between segmentation success of ACWE and SPF, the average success rate of ACWE was approximately 2–4% superior to SPF method in all cases. Although this difference seems small it is important in medical decision making (Table 2), because deformity of the humeral head in Hill-Sachs lesion and location of edema in posttraumatic cases have a peripheric placement in the segmented humeral head which renders slight improvements critical in reaching the correct diagnosis.

The number of iterations and computational time of the ACWE and SPF methods when operated in ROI were 650-23.92 sec and 120-15.72 sec, respectively. Although the computational cost of ACWE method was higher, its success rate was also higher than SPF method. Determination of ROI increased the success of the segmentation by decreasing the studied area and time to the desired limits. A number of iterations decrease by approximately tenfolds by determining ROI area.

The clustering methods of FCM and GMM were able to segment humeral head in normal group with lower success rates than ACWE and SPF. These methods could not provide reasonable results in edematous humeral heads or humeral heads with Hill-Sachs deformity.

## 5. Conclusions

In this study humeral head segmentation was performed on PD weighted axial slices of the shoulder MRI. There is no perfect method to visualize shoulder instability; yet there is a perfect method to segment the humeral head. To overcome the problems of imaging and to increase the success rates of the ACWE and SPF methods preprocessing and postprocessing steps have crucial importance. Our original data set which is composed of patients is therefore a limitation of this study. Manual segmentation with a mouse is the other limitation. There remains some impurities after manual segmentation which may affect the control group data negatively also affecting the final result. However the overall results of our study are still promising. Correct segmentation of humeral head may help to diagnose the humeral head defects which may be a subject of a further study.

## Figures and Tables

**Table 1:** Segmentation results of three groups of humeral heads before and after postprocessing operation.

Methods and data	Average success rate before postprocessing [%]				Average success rate after postprocessing [%]			
	Normal humeral head	Humeral head with edema	Humeral head with Hill-Sachs lesion	Average success rate	Normal humeral head	Humeral head with edema	Humeral head with Hill-Sachs lesion	Average success rate
Number of data	81	100	38	219	81	100	38	219
ACWE method	**90.79**	88.40	86.53	88.95	**92.17**	90.20	**88.90**	90.70
SPF method	**88.42**	87.18	83.25	86.95	**90.30**	89.32	**84.71**	88.88

**Table 2:** Segmentation results obtained by using ACWE, SPF, GMM, and FCM methods.

Methods	Success rate for 81 normal MR images [%]			
	ACWE method	SPF method	GMM method	FCM method
Average success rate	92.17	90.30	83.25	76.83
Maximum segmentation success	97.27	96.12	87.54	83.59
Minimum segmentation success	85.76	82.91	64.39	59.18
